# A Stimuli-Responsive Biosensor of Glucose on Layer-by-Layer Films Assembled through Specific Lectin-Glycoenzyme Recognition

**DOI:** 10.3390/s16040563

**Published:** 2016-04-20

**Authors:** Huiqin Yao, Qianqian Gan, Juan Peng, Shan Huang, Meilin Zhu, Keren Shi

**Affiliations:** 1 Department of Pharmacy, Ningxia Medical University, Yinchuan 750004, China; sarah_ganq@163.com (Q.G.); jay70281@163.com (S.H.); zhanggb@nxmu.edu.cn (M.Z.); 2 State Key Laboratory Cultivation Base of Natural Gas Conversion, Ningxia University, Yinchuan 750021, China; pengjuan@nxu.edu.cn; 3 School of Chemistry and Chemical Engineering, Ningxia University, Yinchuan 750021, China

**Keywords:** stimuli-responsive biosensor, lectin-sugar specific interaction, layer-by-layer assembly, concanavalin A, enzyme immobilization, ferrocenedicarboxylic acid

## Abstract

The research on intelligent bioelectrocatalysis based on stimuli-responsive materials or interfaces is of great significance for biosensors and other bioelectronic devices. In the present work, lectin protein concanavalin A (Con A) and glycoenzyme glucose oxidase (GOD) were assembled into {Con A/GOD}_n_ layer-by-layer (LbL) films by taking advantage of the biospecific lectin-glycoenzyme affinity between them. These film electrodes possess stimuli-responsive properties toward electroactive probes such as ferrocenedicarboxylic acid (Fc(COOH)_2_) by modulating the surrounding pH. The CV peak currents of Fc(COOH)_2_ were quite large at pH 4.0 but significantly suppressed at pH 8.0, demonstrating reversible stimuli-responsive on-off behavior. The mechanism of stimuli-responsive property of the films was explored by comparative experiments and attributed to the different electrostatic interaction between the films and the probes at different pH. This stimuli-responsive films could be used to realize active/inactive electrocatalytic oxidation of glucose by GOD in the films and mediated by Fc(COOH)_2_ in solution, which may establish a foundation for fabricating novel stimuli-responsive electrochemical biosensors based on bioelectrocatalysis with immobilized enzymes.

## 1. Introduction

Stimuli-responsive bioelectronic surfaces, which are capable of properties-on-demand changes upon communication with an external signal, have attracted a great deal of academic and industrial attention due to their potential applications in biosensors, drug delivery, bioseparation, microfluidic devices, permselective membrane and so on [1,2,3,4]. In this regard, active/deactive electrochemical biosensors based on bioelectrocatalysis of enzyme reactions and stimuli-responsive films have aroused more and more interest because they can not only be applied to fabricate intelligent bioelectronic devices, but may also provide a basis for biocomputing, information storage/processing, and signal transduction/amplification [5,6,7]. Particularly, the pH controllable bioelectrocatalysis with enzymes is the most studied variant [4,7,8,9,10,11]. For example, Liu and coworkers reported that {PAH/PAA}_n_ layer-by-layer (LbL) films assembled by poly(allylamine hydrochloride) (PAH) and poly(acrylic acid) (PAA) on electrode surfaces showed a reversible pH switchable bioelectrocatalysis [11]. The films exhibited a pH-responsive structure change and could be employed to activate/deactivate the bioelectrocatalysis of glucose by glucose oxidase. However, in most pH-triggered bioelectrocatalysis studies, the catalytic enzymes were dissolved in the solution but not immobilized into the films. It is known that the enzymes dissolved in solution cannot be recycled or reused efficiently. Particularly, the immobilization of enzyme on biosensors is usually a necessary step that can greatly improve the stability of the enzyme. Therefore, the effective immobilization of enzymes on electrodes is highly desirable for the development of pH-triggered biosensors.

Among a variety of methods available for the immobilization of enzymes, the LbL assembly demonstrates notable advantages in the precise control of film thickness at a nanometer scale according to a predesigned architecture and in its extremely simple procedure and high assembly versatility [12]. Initially, the driving force of LbL assembly was just electrostatic interaction between oppositely charged species, but now it has been expanded to many kinds of non-electrostatic interactions such as hydrophobic interaction, hydrogen bonding, ion-dipole interaction and biospecific recognition [13]. Lectin-sugar interaction is a biospecific recognition and has attracted considerable attention in scientific research. Con A is the best known member of the lectins and has been widely used as a representative lectin. Con A exists as a tetramer and each subunit contains a binding site to recognize the −OH group at the C-4 and C-6 sites of sugar molecules such as glucose and mannose, forming a highly specific 1:4 Con A-sugar complex [14,15,16]. For instance, in our previous work [17], Con A and horseradish peroxidase (HRP) with mannose residues on the surface were assembled into {Con A/HRP}_n_ LbL films on electrodes by the biospecific interaction between them. The cyclic voltammetric (CV) response of Fe(CN)_6_^3^^−^ at {Con A/HRP}_n_ film electrodes was very sensitive to the surrounding pH, which could be used to control the pH stimuli-responsive electrocatalytic reduction of H_2_O_2_ by HRP in the films and mediated by Fe(CN)_6_^3^^−^. Because the sensing of glucose is of great significance nowadays in several applications including the clinical diagnosis of diabetes, switchable glucose sensors based on glucose oxidase (GOD) are thus inherently attractive. The glycoenzyme GOD contains many glucose residues on its surface, and can bind to Con A [18]. Herein, the construction of Con A/GOD films is reversible, and the disintegration of the films occurs in solutions with excess “free” glucose, since the GOD combined with Con A in the films could be replaced by glucose under suitable conditions [19,20]. Therefore, Con A and glycoenzyme GOD can be assembled into LbL films designated as {Con A/GOD}_n_ by the biospecific lectin-sugar affinity between them [21]. To the best of our knowledge pH active/deactive bioelectrocatalysis with {Con A/GOD}_n_ LbL films has not been reported. 

In the present work, Con A and GOD were chosen as the building blocks to be assembled into {Con A/GOD}_n_ LbL films mainly because the driving force of the assembly was the specific lectin-glycoenzyme recognition between them. Herein, GOD could act in two roles: one is the building block of the films; at the same time, it is used as the catalyst for the bioelectrocatalysis. The films showed pH-sensitive on-off properties toward the electrochemical responses of the electroactive probe ferrocenedicarboxylic acid (Fc(COOH)_2_). At pH 4.0, the CV response of Fc(COOH)_2_ was quite large at the film electrodes; however, at pH 8.0, the corresponding CV peak current of the probe cannot be detected. The mechanism of the stimuli-responsive properties of the films toward probes was further explored by comparative studies, and could be mainly attributed to the electrostatic interactions between the films and probe. This reversible stimuli-responsive property was further applied to activate/deactivate the electrocatalytic oxidation of glucose catalyzed by GOD immobilized in the films with Fc(COOH)_2_ probe in solution as the mediator. To mimic the biological environment, this stimuli-responsive bioelectrocatalysis of glucose was also realized by *in situ* enzyme-catalyzed reactions. Compare to pH-sensitive chitosan-GOD films [22], which exhibited an “on” state at pH 5.0 and “off” state at pH 9.0, {Con A/GOD}_n_ films have the same pH sensitive range and ΔpH = 4.0. While works on either biospecific interactions between Con A and GOD [18,21] or pH-switchable bioelectrocatalysis of glucose with immobilization of GOD enzymes such as chitosan-GOD film [22] have been reported previously, to the best of our knowledge, studies on combining them together have not been reported up to now. This work provides a novel example of the construction of a pH stimuli-responsive enzyme-immobilized biointerface and intelligent bioelectrocatalysis of glucose based on the lectin-glycoenzyme biospecific LbL assembly, which may find its potential application in designing new kinds of stimuli-responsive biosensors and bioelectronic devices, and also in mechanistic studies of metabolic pathways.

## 2. Experimental Section

### 2.1. Reagents

Poly(diallyldimethylammonium) (PDDA, 20%), concanavalin A extracted from Jack beans (Con A, type V, MW ≈ 104,000), glucose oxidase (GOD, E.C. 1.1.3.4, type VII, MW ≈ 160,000, 192,000 units·g^−^^1^), esterase from porcine liver (Est, 17,000 units·g^−^^1^), urease from Jack beans (Ur, E.C. 3.5.1.5, type III, 39,290 units·g^−^^1^), 1,1′-ferrocenedicarboxylic acid (Fc(COOH)_2_), ferrocenecarboxylic acid (Fc(COOH)), ferrocenemethanol (FcMeOH), tris(hydroxymethyl) aminomethane (Tris), 3-mercapto-1-propanesulfonate (MPS, 90%) and hexaammineruthenium(III) chloride (Ru(NH_3_)_6_Cl_3_) were purchased from Sigma–Aldrich (Beijing, China). Ethyl butyrate, urea, potassium ferricyanide (K_3_Fe(CN)_6_), potassium ferrocyanide (K_4_Fe(CN)_6_), were obtained from Beijing Chemical Engineering Plant (Beijing, China). All the chemicals were reagent-grade and used as received. Buffers were usually 0.05 mol·dm^−3^ sodium acetate (pH 4–6) or 0.05 mol·dm^−3^ potassium dihydrogen phosphate (pH 6.5–8), all containing 0.1 mol·dm^−3^ NaCl. The pH value of buffers was adjusted to the desired value with dilute HCl or NaOH solutions. The d-glucose stock solutions were allowed to mutarotate at room temperature for 24 h before being used. 0.1 M Tris-HCl buffers at pH 7.0 containing 0.1 M NaCl, 1 mM MnCl_2_ and 1 mM CaCl_2_ were used to prepare Con A and GOD solutions [20]. Deionized water (103S Still, High-Q, Inc., Wilmette, IL, USA) with a resistivity of >10.0 MΩ was used in all experiments. 

### 2.2. Film Assembly

For electrochemical study, basal plane pyrolytic graphite (PG) disk electrodes (geometric area 0.16 cm^2^, Advanced Ceramics, Strongsville, Oh, USA) were used as the substrate for film assembly. Prior to assembly, the PG electrodes were abraded on 600-grit metallographic sandpaper while flushing with water. After being sonicated in water for 30 s and dried in air, the electrodes were first immersed in 1 mg·mL^−1^ PDDA aqueous solutions for 20 min, forming a PDDA precursor layer on PG surface. The PG/PDDA electrodes were then alternately immersed into Con A (1 mg·mL^−1^, pH 7.0) and GOD (1 mg·mL^−1^, pH 7.0) solutions for 20 min each, with intermediate water washing for about 10 s and air stream drying until the desired number of bilayers (*n*) was obtained, forming {Con A/GOD}_n_ LbL films on the PG/PDDA surface.

Quartz crystal microbalance (QCM) measurements were used to confirm film assembly. A gold-coated quartz crystal resonator (International Crystal Manufacturing Co., Oklahoma City, OK, USA) was first covered by a few drops of a freshly prepared “piranha” solution on each side for 10 min, and then washed thoroughly with water and ethanol successively. The QCM gold electrodes were then immersed in 4 mM MPS ethanol solutions for 24 h to chemisorb an MPS monolayer on gold surface by formation of Au-S bond between Au and MPS, introducing negative charges on the surface. The PDDA precursor layer and following {Con A/GOD}_n_ LbL films were then assembled on Au/MPS surface in the same way as on the PG electrodes. 

### 2.3. Apparatus and Procedures

A CHI 660A electrochemical workstation (CH Instruments, Shanghai, China) was used for electrochemical measurements. A typical three-electrode cell was used with a saturated calomel electrode (SCE) as the reference, a platinum wire as counter electrode, and the PG disk electrode with films as the working electrode. The solution was purged with high-purity nitrogen for at least 10 min before electrochemical measurements. The nitrogen atmosphere was then kept above the cell for the entire experiment. Electrochemical impedance spectroscopy (EIS) measurements were performed in 1:1 K_4_Fe(CN)_6_:K_3_Fe(CN)_6_ mixture solutions with total concentration of 10 mM, and a sinusoidal potential modulation with amplitude of ±5 mV and frequency from 10^5^ to 0.1 Hz was superimposed on the formal potential of Fe(CN)_6_^4^^−/3^^−^ redox couple at 0.17 V *vs.* SCE.

QCM was carried out with a CHI 420 electrochemical analyzer (CH Instruments). The quartz crystal resonator (AT-cut) has a fundamental resonance frequency of 8 MHz and is covered by thin gold films on both sides (geometric area 0.196 cm^2^ per one side). After each adsorption step, the QCM gold electrodes were washed thoroughly in water, dried under a nitrogen stream, and the frequency change was then measured in air by QCM.

The pH measurements were performed with PHSJ-3F pH-meter (Shanghai Precision & Scientific Instruments, Shanghai, China). The surface SEM of the films was obtained by an S-4800 scanning electron microscope (Hitachi, Tokyo, Japan) with an acceleration voltage of 5 kV. The {Con A/GOD}_5_ films assembled on PG/PDDA electrodes were used as the SEM sample. Before SEM imaging, the sample surface was coated by thin platinum films with an E-1045 sputtering coater (Hitachi).

## 3. Results and Discussion

### 3.1. Fabrication of {Con A/GOD}_n_ LbL Films

**Scheme 1:**
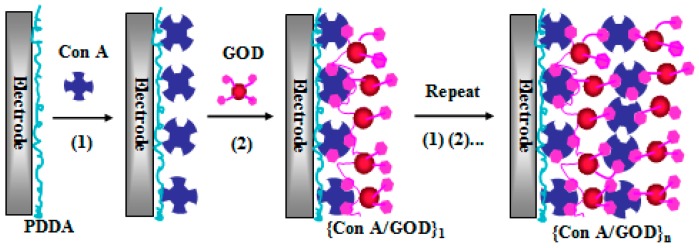
Schematic picture of the {Con A/GOD}_n_ LbL film assembly.

After the bare PG electrodes were abraded by sandpaper, the “edge” surface of rough basal plane with hydrophobic character was uncovered, leading to carry negatively charged by virtue of the surface oxygen functionalities [23]. PDDA are strong polybases and carry positive charges. Thus, PDDA can be adsorbed on PG solid substrates act as a precursor layer. With its isoelectric point at around 5.0 [24], Con A carrying net negative surface charges at pH 7.0 adsorbed on the oppositely charged PG/PDDA surface. The main driving force between PG and PDDA, and between PDDA and Con A should be electrostatic interaction. As the best known lectin protein, Con A possesses a strong biospecific affinity with sugar groups [14,15,16]. The glycoenzyme GOD contains many glucose residues on the surface that can bind to Con A. Thus, Con A and GOD can be alternately assembled into LbL films on the PG/PDDA surface. By repeating this assembly cycle n times, the assembly of {Con A/GOD}_n_ LbL films would be realized mainly by the biospecific lectin-sugar affinity between them (Scheme 1).

**Figure 1:**
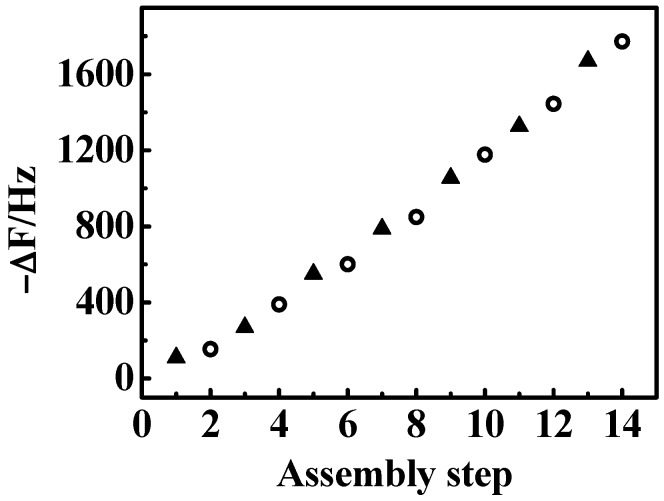
QCM frequency shift (−ΔF) with assembly step of {Con A/GOD}_n_ LbL films on Au/MPS/PDDA surface with adsorption steps: Con A (▲) and GOD (○).

The growth of {Con A/GOD}_n_ LbL films on PG/PDDA surface was first confirmed by QCM (Figure 1). The QCM resonance frequency decreased (−ΔF, Hz) with increased number of layers, reflecting a reproducible mass increase on QCM gold electrodes at each film assembly step. The −ΔF value had a roughly linear relationship with adsorption step, indicating that the {Con A/GOD}_n_ multilayer films are successfully assembled in a regular and reproducible manner. Assembled mass/area (M/A) of each deposited, dried layer of Con A and GOD was obtained from the frequency change (ΔF) on the QCM resonator using the Sauerbrey equation [25]:

M/A (g cm^−2^) = −ΔF (Hz)/(1.83 × 10^8^) (1)

A 1 Hz frequency decrease corresponds to a 0.87 ng mass increase. Assuming that the adsorption layer was densely packed, nominal thickness (d, cm) of each adsorption layer for films deposited on one side of the resonator was estimated according to the following equation [26]:

d = −(3.4 × 10^−9^)ΔF/ρ (2)

where ρ is the density of the layer material (g·cm^−3^). For Con A and GOD, the average density is about 1.3 ± 0.1 g·cm^−3^ [27]. The average frequency decrease for each layer of Con A and GOD are estimated to be 82 ± 25 and 58 ± 20 Hz, respectively, and the corresponding nominal thickness of each layer was 2.1 ± 0.7 nm for Con A and 1.5 nm ± 0.5 nm for GOD.

**Figure 2:**
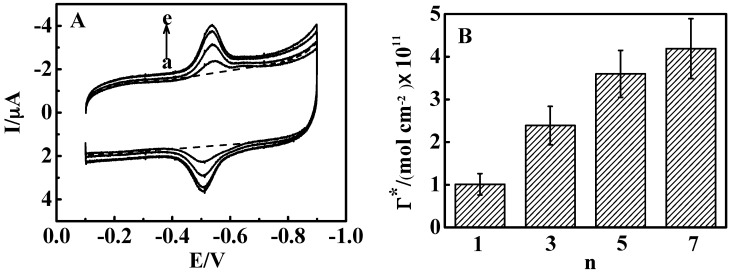
(**A**) CVs in pH 7.0 buffers at 0.2 V·s^−^^1^ for {Con A/GOD}_n_ films assembled on PG/PDDA electrode with different number of bilayers (n): (**a**) 0, (**b**) 1, (**c**) 3, (**d**) 5 and (**e**) 7; (**B**) Effect of number of bilayers on the surface concentration of GOD (Γ*) for {Con A/GOD}_n_.

The assembly of {Con A/GOD}_n_ LbL films was further confirmed by the direct electrochemical response of GOD (Figure 2). For example, the {Con A/GOD}_n_ film electrodes with *n* = 1, 3, 5, 7 were placed into blank pH 7.0 buffers and tested by CV between +0.1 and −0.9 V. A well-defined, nearly reversible reduction-oxidation peak pair was observed at about −0.5 V *vs.* SCE (Figure 2A), characteristic of GOD FAD/FADH_2_ redox couple [28,29,30]. The reduction peak currents demonstrated linear relationship with scan rates from 0.05 to 2.0 V·s^−1^, suggesting that the voltammetric behavior of GOD in the films is diffusionless and thin-layer confined. In this case, integration of CV reduction peak would give the charge (Q) value representing the full reduction of all electroactive GOD in the films. The Q value could be further converted to the surface concentration of electroactive GOD (Γ*, mol·cm^−2^) in the films according to Faraday’s law [31] (Table S1). Γ* increased with the number of bilayers (n) and reached the steady state when *n* = 7 (Figure 2B), and final Γ* is about 4 times of the value of *n* = 1, indicating that the {Con A/GOD}_n_ LbL films are successfully assembled on the PG/PDDA surface.

To confirm film stability, the {Con A/GOD}_5_ films was examined by CV with Fe(CN)_6_^3−^ as the probe. The films were stored in pH 7.0 blank for one week, and then placed in Fe(CN)_6_^3−^ solutions at pH 7.0 for CV testing. The peak potentials maintained the same position, and the peak currents remained nearly the same as their initial values, suggesting that the films are quite stable. The pI of GOD is 4.2 [32]. Thus, in pH 7.0 buffer solutions, both Con A and GOD carry net negative surface charges and would repel one another. However the films could not disintegrate because the main driving force to combine Con A and the GOD is the lectin-sugar biospecific affinity, which can overcome the electrostatic repulsion. This is one of the advantages of {Con A/GOD}_n_ films over other electrostatic LbL films, since the net charge of the films can be easily modulated by the surrounding pH without losing the film stability.

### 3.2. Stimuli-Responsive Behavior of {Con A/GOD}_5_ Films Controlled by Solution pH

**Figure 3:**
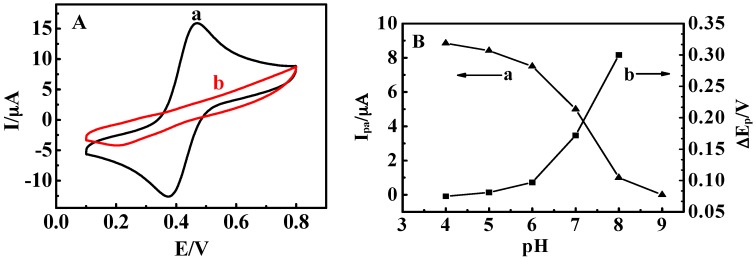
(**A**) CVs of 0.5 mM Fc(COOH)_2_ at 0.1 V·s^−^^1^ for {Con A/GOD}_5_ films in buffers at pH (**a**) 4.0 and (**b**) 8.0; (**B**) Influence of solution pH on (**a**) CV oxidation peak current (I_pa_) and (**b**) peak separation (ΔE_p_) of 0.5 mM Fc(COOH)_2_ at 0.1 V·s^−^^1^ for {Con A/GOD}_5_ films.

For {Con A/GOD}_5_ films with *n* = 5, the solution pH had great influence on the CV behaviors of Fc(COOH)_2_ (Figure 3). For instance, at pH 4.0, Fc(COOH)_2_ displayed a well-defined and nearly reversible CV oxidation-reduction peak pairs at about 0.4 V with quite large peak currents and quite small peak separation (ΔE_p_) (Figure 3A, curve a), and peak currents showed a linear relationship with square root of scan rates from 0.01 to 1.0 V·s^−1^ (Figure S1), suggesting that the electrode reaction of the probe is a diffusion-controlled process. However, at pH 8.0, the CV signals were greatly suppressed and could even hardly be observed (Figure 3A, curve b). From pH 4.0 to 8.0, the CV oxidation peak currents (I_pa_) and ΔE_p_ experienced a dramatic change (Figure S2 and Figure 3B). In control experiments, the CV behavior of Fc(COOH)_2_ at bare PG electrodes was pH-independent (Figure S3). Thus, this pH-sensitive CV behavior of Fc(COOH)_2_ must be related to the interaction between the probe and the films.

**Figure 4:**
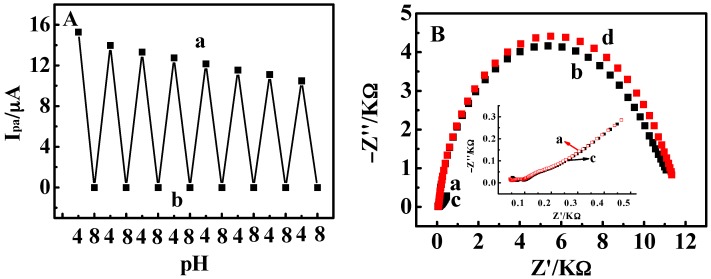
(**A**) Dependence of CV oxidation peak current (I_pa_) of Fc(COOH)_2_ at 0.1 V·s^−^^1^ on solution pH switched between pH 4.0 and 8.0 for the same {Con A/GOD}_5_ films; (**B**) EIS responses of 10 mM Fe(CN)_6_^3−/4−^ at 0.17 V on solution pH switched between (**a**) pH 4.0, (**b**) 8.0 of the first cycle, (**c**) pH 4.0 and (**d**) 8.0 of the second cycle for {Con A/GOD}_5_ films. Inset is a magnification of curve a and curve c.

These results inspired us to use the {Con A/GOD}_5_ films as a pH-triggered switch interface. The {Con A/GOD}_5_ films for Fc(COOH)_2_ were in the “on” state at pH 4.0 and at the “off” state at pH 8.0. This pH stimuli-responsive property of the system was reversible. By switching the film electrode in Fc(COOH)_2_ solutions between pH 4.0 and 8.0, the I_pa_ was changed periodically between the on and off states many times (Figure 4A). In addition, the response rate of the system with pH was very quick, and less than 10 seconds were needed to reach the steady state of CV when the pH of the solution was switched, indicating that the films are very sensitive to surrounding pH. The pH stimuli-responsive behavior of {Con A/GOD}_5_ films toward probe was also observed by EIS (Figure 4B). In pH 4.0 probe buffers, the EIS response in the form of Nyquist diagram exhibited a Warburg line, implying that the charge transfer resistance of the system is very small. However, at pH 8.0, a large semicircle in the high-frequency domain was observed, reflecting the large charge transfer resistance for the system and the films blocked or limited the access of the probe to the electrode surface. This pH-dependent EIS behavior was also reversible between pH 4.0 and 8.0, and could be repeated for several times.

**Figure 5:**
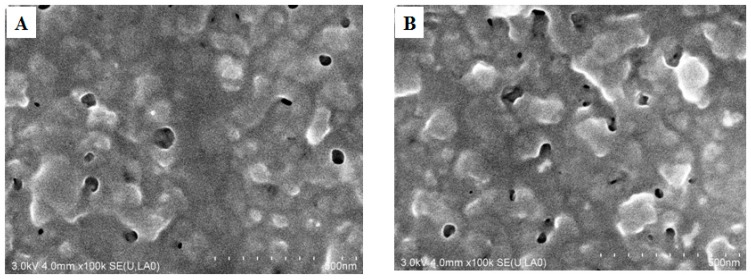
SEM top views of {Con A/GOD}_5_ films assembled on PG/PDDA surface after the films were immersed in buffers containing 0.5 mM Fc(COOH)_2_ at pH (**A**) 4.0 and (**B**) 8.0 for 30 min, and there is no substantial difference in the structures.

This pH stimuli-responsive characteristic of the {Con A/GOD}_5_ films toward Fc(COOH)_2_ can be explained by the electrostatic interaction between the films and the probe. Fc(COOH)_2_ is a weak acid and possesses negatively charges at pH ≥ 4.0 due to its ionization [33]. At pH 8.0, The films carried positive charges since the pIs of Con A and GOD are at about 5.0 [24] and 4.2 [32], respectively, and have a electrostatic repulsion with Fc(COOH)_2_. The strong repulsion between the {Con A/GOD}_5_ films and Fc(COOH)_2_ molecules at this pH hindered the probe from entering the films and restricted the electron exchange of the probe with underlying electrodes, thus resulting in the “off” state of the films. In contrast, at pH 4.0, the films carried net positive charges and have an electrostatic attraction with the probe, leading to the probe to pass through the films very easily and a large probe CV signal. The SEM results showed this pH-triggered stimuli-responsive property have little relationship with the {Con A/GOD}_5_ film structure (Figure 5). The films treated with pH 4.0 buffers showed the same surface morphology as those treated with pH 8.0 buffers, demonstrating that the solution pH has no substantial influence on the structure of the films at least with the present magnification. These results confirm that electrostatic interaction between the films and the probe plays a key role for the stimuli responsive behavior of the films.

**Figure 6:**
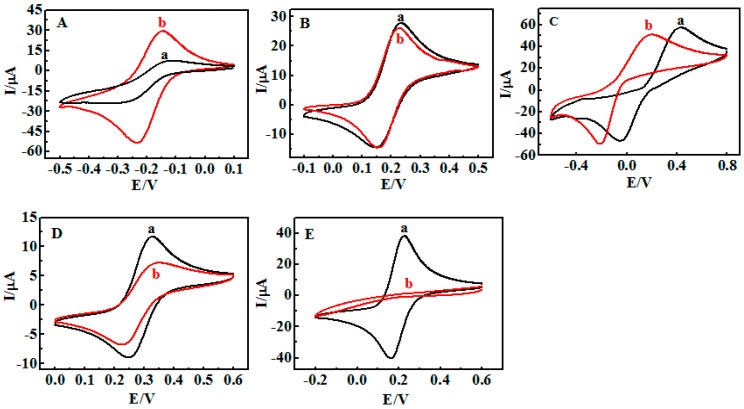
CVs of (**A**) 1 mM Ru(NH_3_)_6_Cl_3_; (**B**) 0.5 mM FcMeOH; (**C**) 0.5 mM hydroquinone; (**D**) 0.2 mM Fc(COOH); and (**E**) K_3_Fe(CN)_6_ at 0.1 V·s^−1^ for {Con A/GOD}_5_ films in buffers at pH (**a**) 4.0 and (**b**) 8.0.

To further support our speculation, three types of probes with different charges were investigated and compared (Figure 6), including positively charged Ru(NH_3_)_6_^3+^ probe, neutral FcMeOH and hydroquinone, and other negatively charged Fe(CN)_6_^3−^ and Fc(COOH) probes. For positively charged Ru(NH_3_)_6_^3+^ probe, its stimuli-responsive CV behavior of pH at {Con A/GOD}_5_ films was direct opposite of negatively charged Fc(COOH)_2_ (Figure 6A). That is, Ru(NH_3_)_6_^3+^ displayed a quite large CV peaks at pH 8.0 and the peaks were greatly suppressed at pH 4.0. For neutral probe FcMeOH (Figure 6B) and hydroquinone(Figure 6C), no essential difference in CV peak currents was observed at pH 4.0 and 8.0. For other negatively charged ferrocenecarboxylic acid (Fc(COOH)) probe (Figure 6D) and Fe(CN)_6_^3−^ probe (Figure 6E), its pH-triggered CV results are similar to those of Fc(COOH)_2_. Therefore, it can be concluded that the stimuli-responsive property of Fc(COOH)_2_ for the {Con A/GOD) films was mainly attributed to the electrostatic interaction between the films and the probe in solution.

### 3.3. Influence of Film Thickness

The influence of the film thickness modulated by the number of bilayers (n) of {Con A/GOD}_5_ films on the stimuli-responsive property was examined by CV. For {Con A/GOD}_1_ films with *n* = 1, the CV peak current of Fc(COOH)_2_ at pH 4.0 was a little higher than that at pH 8.0, but the peak current at pH 8.0 was still quite large (Figure S4). This is understandable since one Con A/GOD bilayer carries relatively small amounts of negative charge at pH 8.0, which had weaker electrostatic attraction with Fc(COOH)_2_. With the increase of n bilayers for the films, the I_pa_ of Fc(COOH)_2_ gradually decreased at both pH 4.0 and 8.0, implying that the permeability of the films toward Fc(COOH)_2_ became poorer with thicker films. Particularly at pH 8.0, the I_pa_ decreased dramatically from *n* = 1 to 5, and the peak disappeared when *n* ≥ 5. This is because the thicker films carry larger amounts of negative charges at this pH, leading to the stronger electrostatic repulsion with Fc(COOH)_2_. The most pronounced difference in I_pa_ values between pH 4.0 and 8.0 was observed with *n* = 5 films, the {Con A/GOD}_5_ films were thus used in this work.

The influence of outermost layer of {Con A/GOD}_n_ films was also investigated (Figure S5). For example, under the same conditions, {Con A/GOD}_4_-Con A and {Con A/GOD}_5_ films exhibited nearly identical CV stimuli-responsive responses, suggesting the interpenetration of neighboring layers in the assembly, which is a common phenomenon in LbL assembly [34].

### 3.4. Stimuli-Responsive Bioelectrocatalysis for {Con A/GOD}_5_ Films

**Figure 7:**
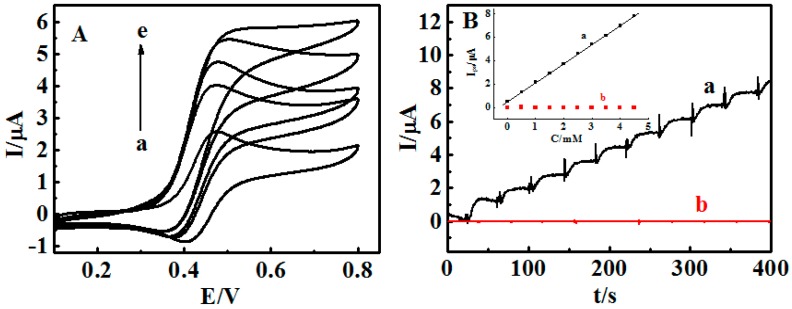
(**A**) CVs of {Con A/GOD}_5_ films at 0.005 V·s^−1^ in pH 4.0 buffers containing 0.5 mM Fc(COOH)_2_ with (**a**) 0, (**b**) 3.0, (**c**) 5.5, (**d**) 6.5, and (**e**) 7.0 mM glucose; (**B**) Amperometric responses of {Con A/GOD}_5_ films at 0.4 V in buffers containing 0.5 mM Fc(COOH)_2_ upon successive addition of 0.5 mM glucose at pH (**a**) 4.0 and (**b**) 8.0. Inset: dependence of amperometric oxidation peak currents (I_pa_) on concentration of glucose in systems at pH (**a**) 4.0 and (**b**) 8.0.

**Figure 8:**
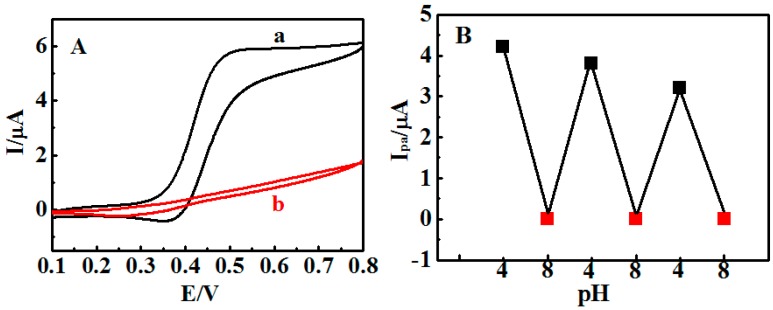
(**A**) CVs of {Con A/GOD}_5_ films at 0.005 V·s^−1^ in solutions containing 0.5 mM Fc(COOH)_2_, 7.0 mM glucose at pH (**a**) 4.0 and (**b**) 8.0; (**B**) Dependence of CV catalytic I_pa_ at 0.005 V·s^−1^ on solution pH switched between pH 4.0 and 8.0, which tuned by alternate addition of 10 mM ethyl butyrate (black square) and 6 mM urea (red square) into unbuffered solutions containing 5 units·mL^−1^ esterase, 15 units·mL^−1^ urease, 0.5 mM Fc(COOH)_2_, and 7.0 mM glucose for the same {Con A/GOD}_5_ films.

The pH stimuli-responsive property of {Con A/GOD}_5_ films toward Fc(COOH)_2_ probe can be used to modulate the bioelectrocatalytic oxidation of glucose. For example, when the {Con A/GOD}_5_ film electrode was placed in a solution containing Fc(COOH)_2_ and glucose at pH 4.0, in comparison with the system without glucose, an obvious increase of the CV oxidation peak current was observed, accompanied by the decrease or even disappearance of the reduction peak (Figure 7A). The catalytic oxidation wave current initially increased with the concentration of glucose in solution and then reached its maximum value (Figure S6). The bioelectrocatalysis of glucose at {Con A/GOD}_5_ films was further characterized by amperometry. In pH 4.0 solutions containing Fc(COOH)_2_ probe, with the successive addition of glucose, a stepped increase of amperometric oxidation currents was observed (Figure 7B, curve a). The oxidation currents had a linear relationship with glucose concentration in the range of 0.5–5 mM (Figure 7B, inset). All these are characteristic of the electrochemical oxidation of glucose catalyzed by GOD immobilized in the films and mediated by Fc(COOH)_2_ in solution [35]. However, when {Con A/GOD}_5_ films were placed in pH 8.0 buffers containing the same amount of Fc(COOH)_2_ and glucose, the CV response was too small to be detected (Figure 8A, curve b), in comparison with that at pH 4.0 (Figure 8A, curve a). Similarly, no increase of oxidation current was observed from the amperometric responses at pH 8.0 for the same system (Figure 7B, curve b). Because the bioelectrocatalytic response of the GOD + glucose + Fc(COOH)_2_ system was pH-independent [36,37,38], it is the pH stimuli-responsive property of the {Con A/GOD}_5_ films toward the probe but not the enzymatic reactions that control the on-off bioelectrocatalysis of glucose for the films. This is because the films become “closed” toward Fc(COOH)_2_ at pH 4.0, resulting in the interruption of the catalytic cycles. Therefore, the bioelectrocatalytic reaction of glucose could be controlled by {Con A/GOD}_5_ films at different pH values. The electrocatalysis was “active” at pH 4.0 and “inactive” at pH 8.0. This stimuli-responsive bioelectrocatalysis for the {Con A/GOD}_5_ films was also reversible and could be repeated for several cycles by switching the same films in the Fc(COOH)_2_ + glucose solutions between pH 4.0 and 8.0 (Figure S7).

Theoretically, glucose could also combine with Con A through a similar biospecific interaction and thus would compete with GOD in {Con A/GOD}_5_ films. That is, the {Con A/GOD}_n_ films might disintegrate upon exposure to glucose in solution. However, the association constant between Con A and glucose is only about 8 × 10^2^ M [39,40], and much smaller than the constant (10^5^–10^7^ M^−1^) of Con A with GOD [41,42]. The lectin-sugar biospecific interaction between Con A and glucose is far weaker than that between Con A and GOD, so the competitive reaction of Con A-GOD + glucose → Con A-glucose + GOD may occur when the concentration of glucose is high enough [43,44]. In the present work, the maximum concentration of glucose used was 7.0 mM, much lower than that needed (100 mM) for the disintegration between Con A and glycoenzymes GOD films [20], and the {Con A/GOD}_5_ films were quite stable in the bioelectrocatalytic system. To further confirm the stability of {Con A/GOD}_5_ films in glucose solution, the films were immersed in pH 7.0 buffers containing 7.0 mM glucose. After 20 h of immersion, the CV response of the films was monitored and the surface concentration of electroactive GOD (Γ*) in {Con A/GOD}_5_ films did not decrease, indicating that the films were quite stable under the relatively low concentration of glucose condition employed in the present work.

In real physiological and biological systems, the various pH stimuli-responsive processes are adjusted by different biochemical reactions [45,46]. To mimic the biological environment, we tried to modulate the system pH by *in situ* enzyme catalyzed reactions, which were used in the pH stimuli-responsive bioelectrocatalysis of glucose with the {Con A/GOD}_5_ + Fc(COOH)_2_ system. For example, the {Con A/GOD}_5_ films were placed in unbuffered solutions containing Fc(COOH)_2_, glucose, esterase and urease. After ethyl butyrate was added, the pH value could change from initial 6.5 to 4.0 by the product of butyric acid. Herein, the electrocatalytic oxidation I_pa_ value of glucose was quite large (Figure S8, curve a). Urea was then added into the solution, leading to the solution pH increase up to 8.0 because of ammonia produced by enzymatic catalysis with urease. The CV was scanned again but the electrocatalytic peak could not be observed (Figure S8, curve b). This *in-situ* stimuli-reponsive behavior could also be cycled several times (Figure 8B), which is consistent with the CV results of the pH tuned by artificial addition of sodium hydroxide or hydrochloric acid.

## 4. Conclusions

Based on specific biological interaction between lectin Con A and the glycoenzyme GOD, {Con A/GOD}_n_ LbL films were successfully assembled on various solid surfaces and GOD is immobilized on the electrode surface. The films exhibit pH-dependent stimuli-responsive CV on-off properties toward different charged probes such as Fc(COOH)_2_. This is because the films are stabilized mainly by the unique lectin-sugar rather than electrostatic interaction, and the net charge of the films could be easily tuned by solution pH. A series of comparative experiments confirm that the electrostatic interaction between the films and the probe plays a key role in deciding the stimuli-responsive on-off behavior of the system. Herein, Fc(COOH)_2_ not only acts as the electroactive probe, but also could be used as the mediator for enzymatic electrocatalytic reactions. The {Con A/GOD}_n_ films can be used to switch the electrocatalysis oxidation of glucose catalyzed by GOD in the films by altering the environmental pH. This stimuli-responsive biointerface with immobilized enzymes based on lectin-glycoenzyme specific recognition provides a foundation or model for fabricating new kinds of intelligent electrochemical glucose biosensors or other bioelectronic devices based on bioelectrocatalysis.

## Supplementary Materials

The following are available online at http://www.mdpi.com/1424-8220/16/4/563/s1, Figure S1: (A) CVs of 0.5 mM Fc(COOH)_2_ in pH 4.0 buffers for {Con A/GOD}_5_ films at different scan rates (V·s^−^^1^): (a) 0.01, (b) 0.02, (c) 0.03, (d) 0.04, (e) 0.05, (f) 0.06, (g) 0.07, (h) 0.08, (i) 0.09 and (j) 1.0. (B) Effect of scan rate (*v*) on the I_pa_ of Fc(COOH)_2_ for {Con A/GOD}_5_ films. Figure S2: CVs of 0.5 mM Fc(COOH)_2_ for {Con A/GOD}_5_ films at 0.1 V·s^−^^1^ in buffers at pH (a) 4.0, (b) 4.5, (c) 5.0, (d) 6.0, (e) 7.0, and (f) 8.0. Figure S3: CVs of 0.5 mM Fc(COOH)_2_ at 0.1 V·s^−^^1^ for bare PG electrode in buffers at pH (a) 4.0 and (b) 8.0. Figure S4: Influence of the number of bilayers (n) for {Con A/GOD}_n_ films on CV reduction peak current (I_pa_) of 0.5 mM Fc(COOH)_2_ in buffers at pH 4.0 (○) and 8.0 (●) at 0.1 V·s^−^^1^. Figure S5: CVs of 0.5 mM Fc(COOH)_2_ at 0.1 V·s^−^^1^ for {Con A/GOD}_4_/Con A (red) and {Con A/GOD}_5_ (black) films in buffers at pH (a) 4.0 and (b) 8.0, respectively. Figure S6: Dependence of CV electrocatalytic oxidation peak current (I_pa_) at 0.005 V·s^−^^1^ on concentration of glucose at {Con A/GOD}_5_ film electrodes in pH 4.0 solutions containing 0.5 mM Fc(COOH)_2_ and glucose. Figure S7: Dependence of CV catalytic oxidation peak current (I_pa_) at 0.005 V·s^−^^1^ on solution pH switched between pH 4.0 and 8.0 for the same {Con A/GOD}_5_ films. The solution contained 0.5 mM Fc(COOH)_2_ and 7.0 mM glucose. Figure S8: CVs of {Con A/GOD}_5_ films at 0.005 V·s^−^^1^ in solutions containing 0.5 mM Fc(COOH)_2_, 7.0 mM glucose at pH (a) 4.0 and (b) 8.0, which tuned by alternate addition of 10 mM ethyl butyrate (and 6 mM urea into unbuffered solutions containing 5 units·mL^−1^ esterase, 15 units·mL^−1^ urease.
