# Supplementary material for: A Stimuli-Responsive Biosensor of Glucose on Layer-by-Layer Films Assembled through Specific Lectin-Glycoenzyme Recognition
Source: Sensors (Basel). 2016 Apr 20;16(4):563. doi: 10.3390/s16040563 (PMC4851077; doi:10.3390/s16040563)
Supplement: Supplementary file 1 [file sensors-16-00563-s001.pdf]

# Supplementary Materials: Stimuli-Responsive Biosensor of Glucose on Layer-by-Layer Films Assembled through Specific Lectin-Glycoenzyme Recognition

**Huiqin Yao, Qianqian Gan, Juan Peng, Shan Huang, Meilin Zhu and Keren Shi**

**Table S1.** The surface concentration of GOD ($\Gamma^*$, mol·cm$^{-2}$) for {Con A/GOD}$_n$ with different number of bilayers ($n$).

| {Con A/GOD}$_n$ | Q (C) × 10$^7$ | $\Gamma^*$, (mol·cm$^{-2}$) × 10$^{11}$ |
|---|---|---|
| $n = 1$ | 1.559 | 1.010 |
| $n = 3$ | 3.685 | 2.387 |
| $n = 5$ | 5.555 | 3.598 |
| $n = 7$ | 6.468 | 4.190 |

According to the equation of $Q = NAF\Gamma^*$ based on the Faraday's law, where N is the number of electrons transferred (1), A is the geometric area of PG electrode (0.16 cm$^2$) and F is the Faraday constant (96487 C·mol$^{-1}$).

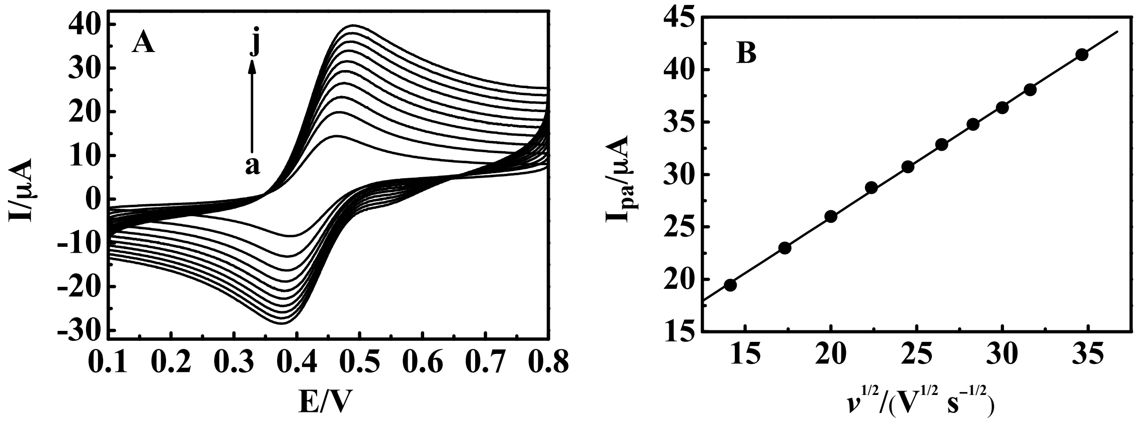


**Figure S1.** (**A**) CVs of 0.5 mM $Fc(COOH)_2$ in pH 4.0 buffers for {Con A/GOD}$_5$ films at different scan rates (V·s$^{-1}$): (**a**) 0.01, (**b**) 0.02, (**c**) 0.03, (**d**) 0.04, (**e**) 0.05, (**f**) 0.06, (**g**) 0.07, (**h**) 0.08, (**i**) 0.09 and (**j**) 1.0; (**B**) Effect of scan rate ($v$) on the $I_{pa}$ of $Fc(COOH)_2$ for {Con A/GOD}$_5$ films.

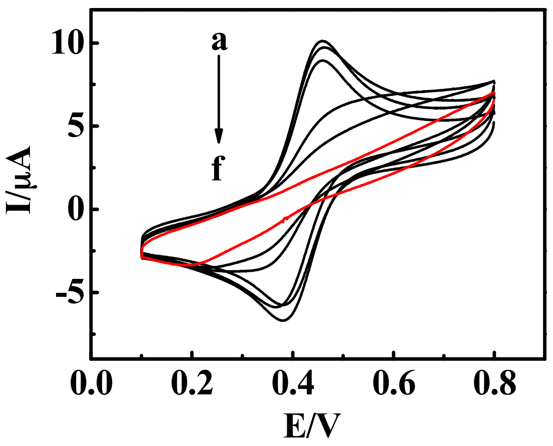


**Figure S2.** CVs of 0.5 mM $Fc(COOH)_2$ for {Con A/GOD}$_5$ films at 0.1 V·s$^{-1}$ in buffers at pH (**a**) 4.0, (**b**) 4.5, (**c**) 5.0, (**d**) 6.0, (**e**) 7.0, and (**f**) 8.0.

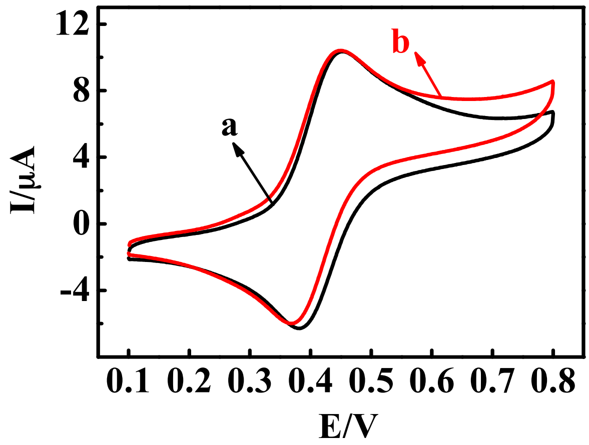


**Figure S3.** CVs of 0.5 mM $Fc(COOH)_2$ at 0.1 $V{\cdot}s^{-1}$ for bare PG electrode in buffers at pH (**a**) 4.0 and (**b**) 8.0.

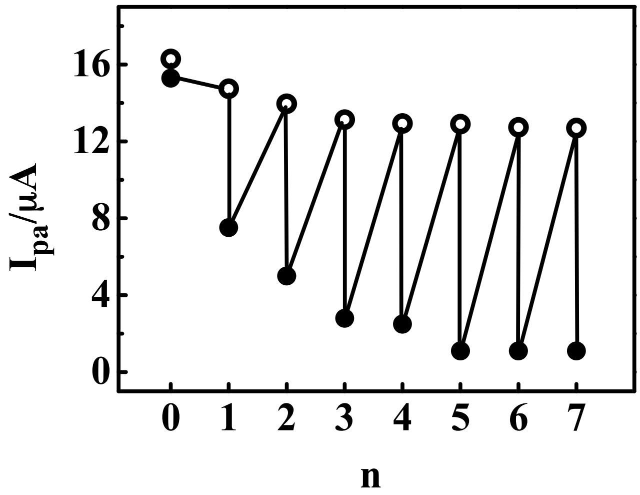


**Figure S4.** Influence of the number of bilayers (*n*) for $\{Con\ A/GOD\}_n$ films on CV reduction peak current ($I_{pa}$) of 0.5 mM $Fc(COOH)_2$ in buffers at pH 4.0 (○) and 8.0 (●) at 0.1 $V{\cdot}s^{-1}$.

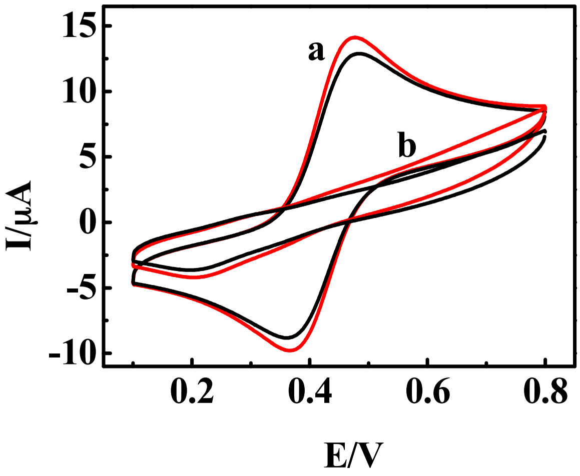


**Figure S5.** CVs of 0.5 mM $Fc(COOH)_2$ at 0.1 $V{\cdot}s^{-1}$ for $\{Con\ A/GOD\}_4$/Con A (red) and $\{Con\ A/GOD\}_5$ (black) films in buffers at pH (**a**) 4.0 and (**b**) 8.0, respectively.

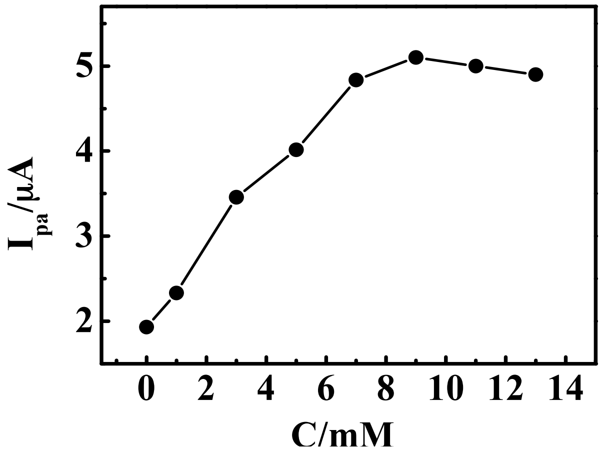


**Figure S6.** Dependence of CV electrocatalytic oxidation peak current ($I_{pa}$) at 0.005 V·s$^{-1}$ on concentration of glucose at {Con A/GOD}$_5$ film electrodes in pH 4.0 solutions containing 0.5 mM $Fc(COOH)_2$ and glucose.

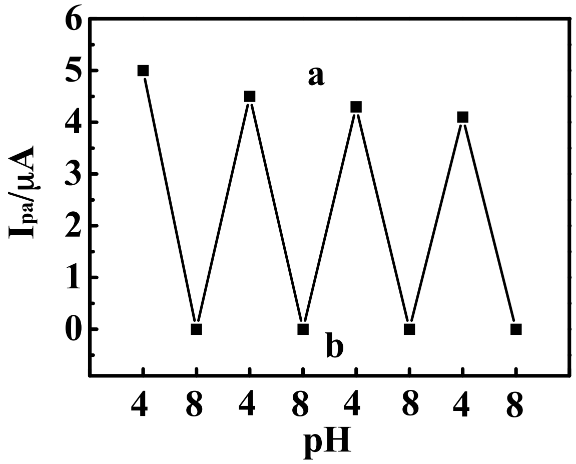


**Figure S7.** Dependence of CV catalytic oxidation peak current ($I_{pa}$) at 0.005 V·s$^{-1}$ on solution pH switched between pH 4.0 and 8.0 for the same {Con A/GOD}$_5$ films. The solution contained 0.5 mM $Fc(COOH)_2$ and 7.0 mM glucose.

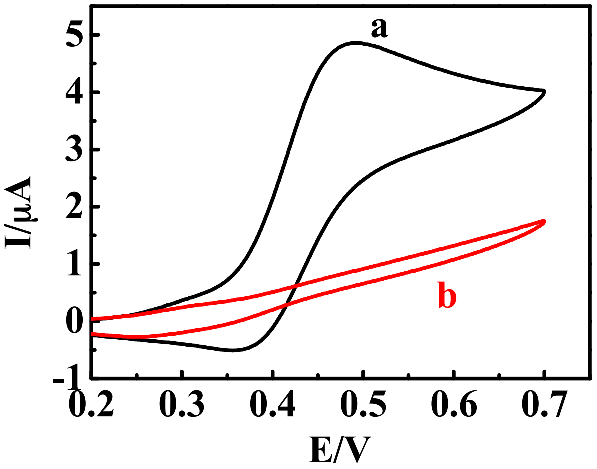


**Figure S8.** CVs of {Con A/GOD}$_5$ films at 0.005 V·s$^{-1}$ in solutions containing 0.5 mM $Fc(COOH)_2$, 7.0 mM glucose at pH (**a**) 4.0 and (**b**) 8.0, which tuned by alternate addition of 10 mM ethyl butyrate and 6 mM urea into unbuffered solutions containing 5 units·mL$^{-1}$ esterase and 15 units·mL$^{-1}$ urease.
